# Sustained year-round oceanographic measurements from Rothera Research Station, Antarctica, 1997–2017

**DOI:** 10.1038/s41597-023-02172-5

**Published:** 2023-05-10

**Authors:** Hugh Venables, Michael P. Meredith, Katharine R. Hendry, Petra ten Hoopen, Helen Peat, Alice Chapman, Jennifer Beaumont, Rayner Piper, Andrew J. Miller, Paul Mann, Helen Rossetti, Ali Massey, Terri Souster, Simon Reeves, Mairi Fenton, Sabrina Heiser, Sam Pountney, Sarah Reed, Zoë Waring, Marlon Clark, Emma Bolton, Ryan Mathews, Hollie London, Alice Clement, Emma Stuart, Aurelia Reichardt, Mark Brandon, Melanie Leng, Carol Arrowsmith, Amber Annett, Sian F. Henley, Andrew Clarke

**Affiliations:** 1 British Antarctic Survey, High Cross, Madingley Road, Cambridge, CB3 0ET UK; 2 Previously affiliated with: British Antarctic Survey, High Cross, Madingley Road, Cambridge, CB3 0ET UK; 3 National Institute of Water and Atmospheric Research (NIWA), Wellington, New Zealand; 4 Fathom Ecology, 39 Maisemore Gardens, Emsworth, Hampshire, England PO10 7JX UK; 5 National Institute of Water and Atmospheric Research (NIWA), Christchurch, New Zealand; 6 Department of Geography and Environmental Sciences, Northumbria University, Ellison Place, Newcastle upon Tyne, NE1 8ST UK; 7 The Norwegian College of Fishery Science, UiT The Arctic University of Norway, 9037 Tromsø, Norway; 8 The Lyell Centre, Heriot-Watt University, Edinburgh, EH14 4AS UK; 9 Marine Science Institute, University of Texas at Austin, Port Aransas, Texas USA; 10 Ocean Ecology Limited - Scottish Office, European Marine Science Park, Malin House, Dunbeg, Oban, PA37 1SZ UK; 11 Basecamp Research, Unit 510 Clerkenwell Workshops, 27 Clerkenwell Close, London, EC1R 0AT UK; 12 School of Environment, Earth and Ecosystem Sciences, The Open University, Walton Hall, Milton Keynes, MK7 6AA UK; 13 Centre for Environmental Geochemistry, School of Biosciences, Sutton Bonington Campus, University of Nottingham, Loughborough, LE12 5RD UK; 14 British Geological Survey, Environmental Science Centre, Nicker Hill, Keyworth, Nottingham, NG12 5GG UK; 15 School of Ocean and Earth Science, University of Southampton, European Way, Southampton, SO14 3ZH UK; 16 School of GeoSciences, James Hutton Road, The University of Edinburgh, Edinburgh, EH9 3FE UK

**Keywords:** Ocean sciences, Physical oceanography, Marine chemistry

## Abstract

Oceanographic changes adjacent to Antarctica have global climatic and ecological impacts. However, this is the most challenging place in the world to obtain marine data due to its remoteness and inhospitable nature, especially in winter. Here, we present more than 2000 Conductivity-Temperature-Depth (CTD) profiles and associated water sample data collected with (almost uniquely) full year-round coverage from the British Antarctic Survey Rothera Research Station at the west Antarctic Peninsula. Sampling is conducted from a small boat or a sled, depending on the sea ice conditions. When conditions allow, sampling is twice weekly in summer and weekly in winter, with profiling to nominally 500 m and with discrete water samples taken at 15 m water depth. Daily observations are made of the sea ice conditions in the area. This paper presents the first 20 years of data collection, 1997-2017. This time series represents a unique and valuable resource for investigations of the high-latitude ocean’s role in climate change, ocean/ice interactions, and marine biogeochemistry and carbon drawdown.

## Background & Summary

The seas around Antarctica are globally important, being intimately connected to the rest of the Earth System. Processes on the Antarctic shelves lead to the formation of the densest waters that participate in the global ocean overturning circulation, and hence play a large role in setting planetary climate^1,2^. The water column is highly biologically productive due to the input of micronutrients from sediments and glaciers, in addition to the high macronutrient concentrations from the upwelling of circumpolar deep water; this structures the marine ecosystem and plays a leading role in ocean carbon cycling^3,4^. These oceans lie adjacent to, and beneath, Antarctic ice shelves and marine terminating glaciers, which are being melted by ocean heat, with consequences for sea level rise globally^5–7^. It is thus of key importance to obtain sustained oceanographic measurements here, however this is especially challenging due to the remoteness and inhospitable nature of the environment. This is particularly the case during the austral winter, when extensive sea ice cover and shortened daylength (with up to 24-hour darkness) hamper operations. As such, year-round observations have great scientific value, given the systematic seasonal biases in nearly all existing datasets.

**Fig. 1:**
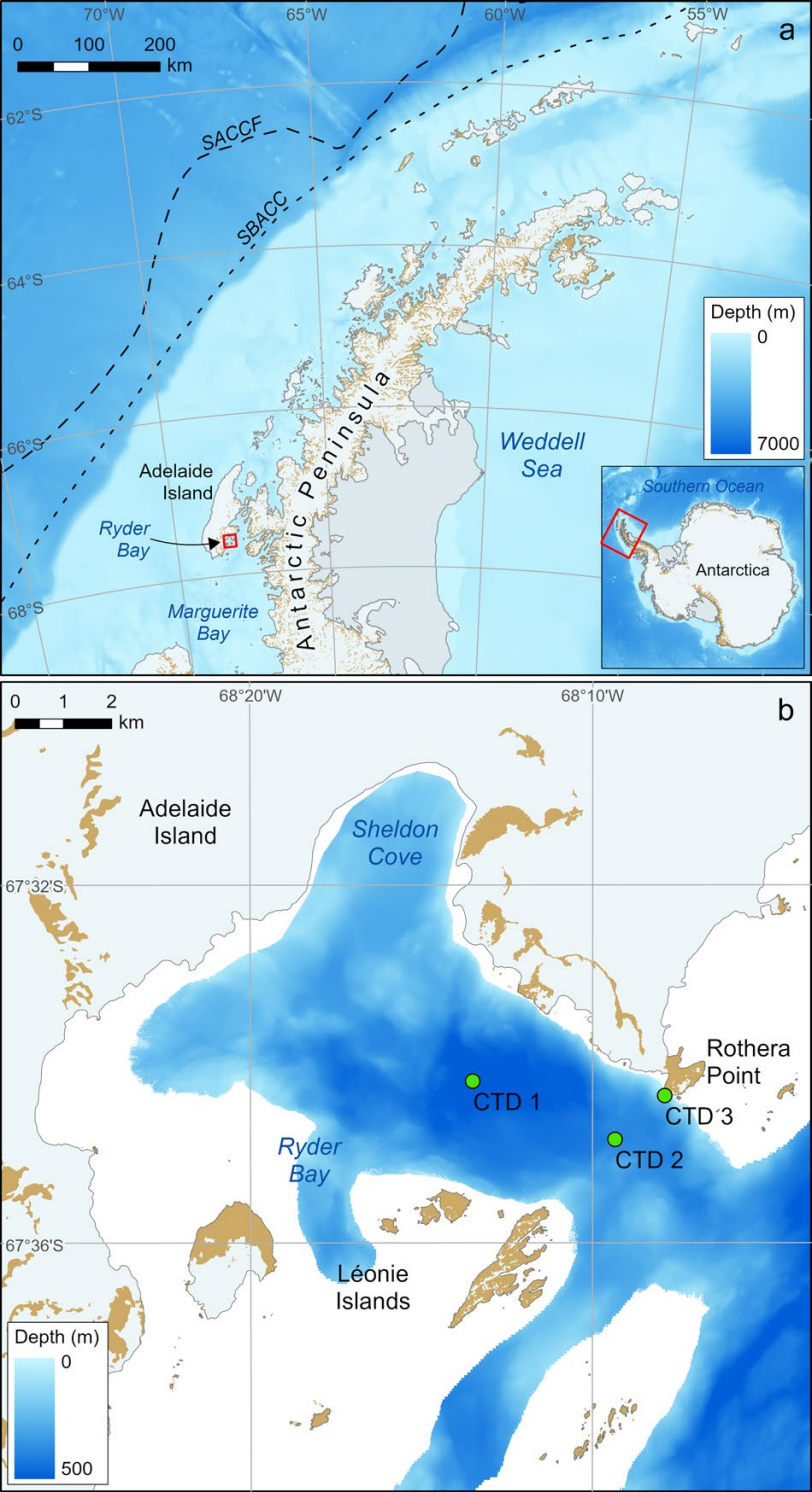
Sampling locations. (**a**) location of Ryder Bay within Marguerite Bay at the west Antarctic Peninsula, WAP; (**b**) sampling sites within Ryder Bay used in Rothera Time Series (CTD sites 1, 2, and 3 shown by green circles). SACCF = Southern Antarctic Circumpolar Current Front; SBACC = Southern Boundary of the Antarctic Circumpolar Current.

Here we report such a year-round dataset collected at Rothera Research Station, which is located adjacent to Ryder Bay, and embayment within Marguerite Bay, at 67°34′8″*S*, 68°7′29″*W* on the eastern side of Adelaide Island at the west Antarctic Peninsula (WAP; Fig. 1). It is now the principal base of the British Antarctic Survey (BAS), with both a deep-water wharf and, since 1992, a 900 m gravel runway. The focus of marine science sampling was relocated to Rothera from Signy Research Station on the South Orkney Islands (60°43′0″*S*, 45°36′0″*W*) following the completion of the runway and the start of regular flights to Rothera from South America and the Falkland Islands in 1994. This led to the inception of the Rothera Oceanographic and Biological Time Series (RaTS) in 1997^8^. The first twenty years of oceanographic and water sampling data from RaTS are presented here. Observations are ongoing, with the intention that this dataset will be updated on a five-yearly basis.

**Fig. 2:**
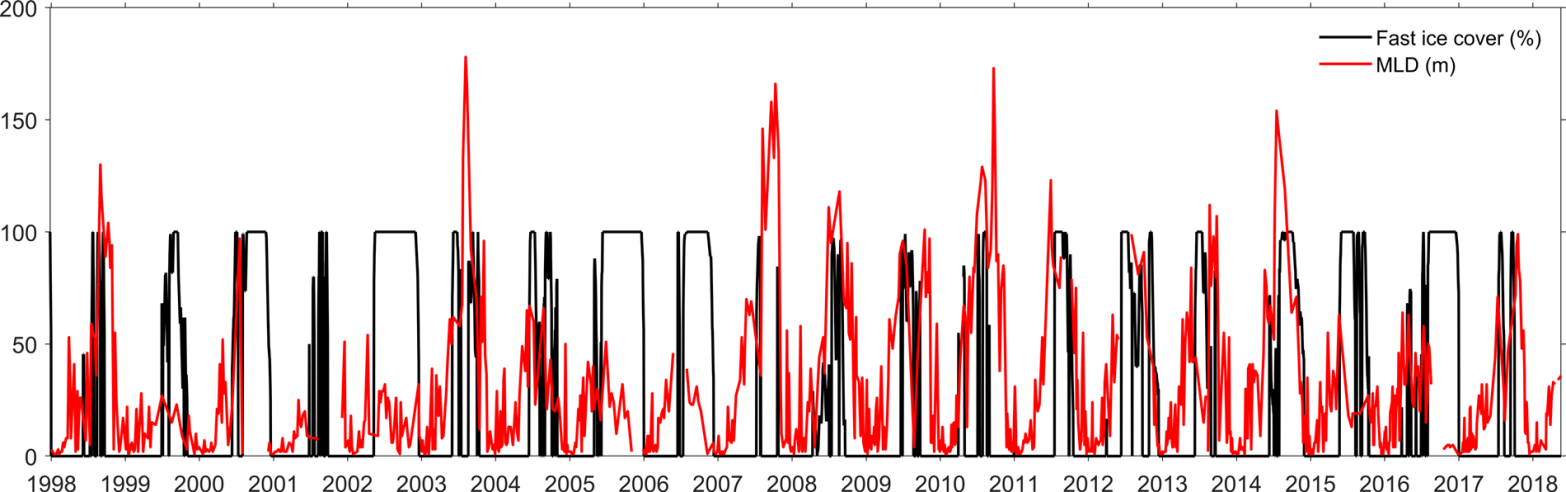
The time series of mixed layer depth (MLD, red line) at the RaTS site and fast ice cover in Ryder Bay (black line), measured as part of the RaTS program.

The WAP has a relatively mild and maritime climate by Antarctic standards, with temperatures above freezing for much of summer and falling to around −20 °*C* in winter. Sea ice forms in winter but is not always present or persistent, depending on variability in air temperature and wind strength and direction. Deep waters are relatively warm (≈1 °*C*) and some of this heat can be vented to the atmosphere, especially in winters with low sea ice cover and hence sustained periods where deep mixing and air-sea fluxes can persist (Fig. 2). These factors combine to create significant variability through the time series on seasonal and interannual timescales.

The atmosphere of the WAP has warmed strongly over the past several decades^9^, though with an apparent hiatus since around the turn of the century. The summertime surface ocean and deep ocean have warmed significantly since the middle of the last century through to the year 2000, with strong impacts on glacial retreat^5,10^. The two decades of data presented here span a significant part of this period, though quantification and investigation of potential trends will be pursued in separate publications.

The deep waters on the WAP shelf are modified Circumpolar Deep Water (mCDW). This derives from the CDW that flows along the shelf break at the southern edge of the Antarctic Circumpolar Current (ACC); this crosses the physical and dynamical barrier of the shelf break to flow onto the WAP shelf. The water reaching the area around Rothera is particularly strongly modified due to the convoluted route through narrow deep channels, with sills blocking the densest water and creating localised mixing due to the overflows^11^. The sill depth into Ryder Bay is 350 m, thus water below these depths will have previously overflowed the sill and likely entrained some overlying water as it does so, leading to some seasonal and interannual variability in deep water properties.

**Fig. 3:**
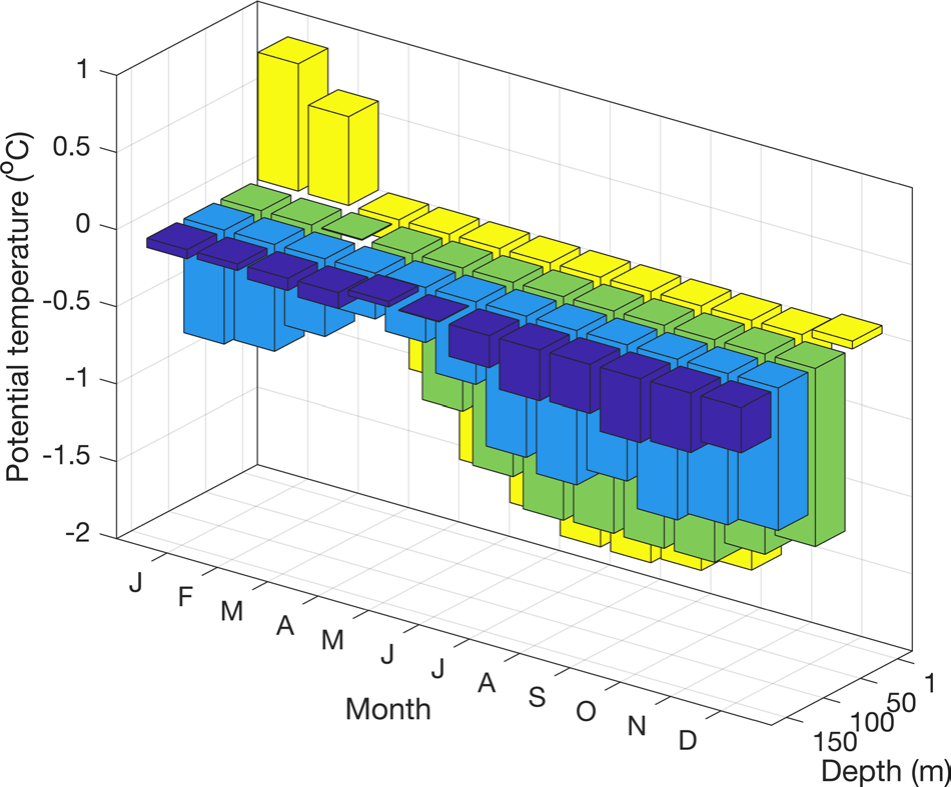
Seasonal climatology of potential temperature measurements at different depth levels (1, 50, 100, and 150 m) from the RaTS dataset (1997–2017).

**Fig. 4:**
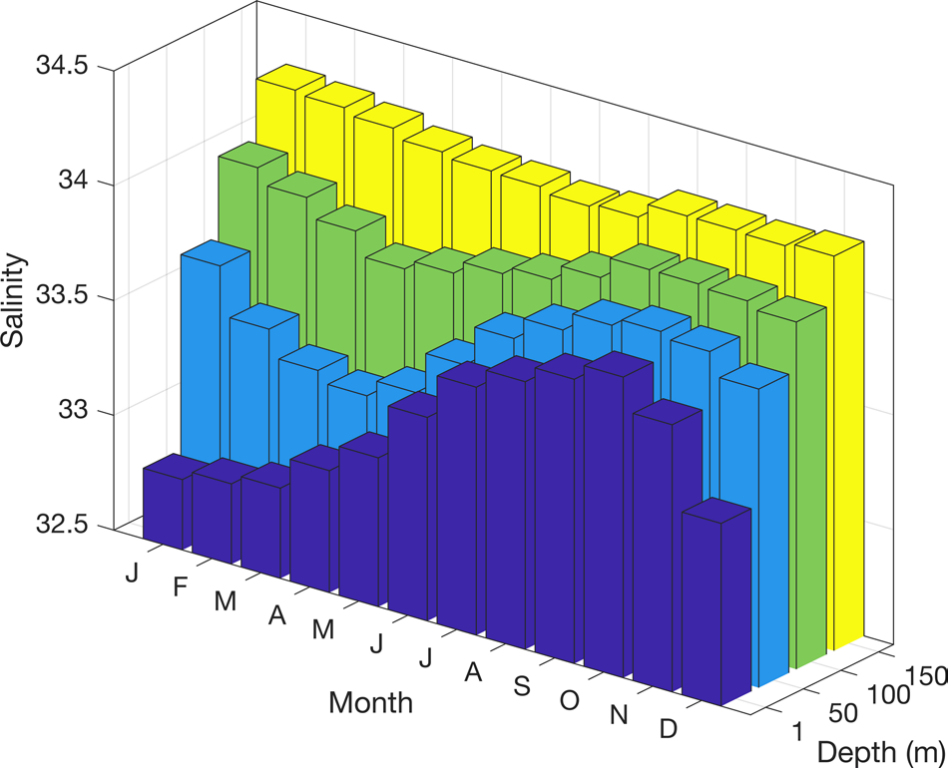
Seasonal climatology of practical salinity measurements at different depth levels (1, 50, 100, and 150 m) from the RaTS dataset (1997–2017). Note depth axis reversed relative to Fig. 3.

Whilst these deeper waters (~150 m and below) show relatively little seasonal variability in temperature and salinity (Figs. 3, 4), the overlying waters show pronounced seasonal changes due to wind-driven and convective mixing in winter. This process creates a deep winter mixed layer (50–100 m thick), which is overlaid in summer with waters that are warmed by insolation and freshened by ice melt. Interannual changes in the depth of winter mixing, often tied to sea ice concentration in winter, result in year-on-year changes in near-surface water mass properties. Low sea ice years have deeper mixed layer depths due to increased wind-driven mixing^12^, with less sea ice formation due to relatively warm air temperatures^13^.

**Fig. 5:**
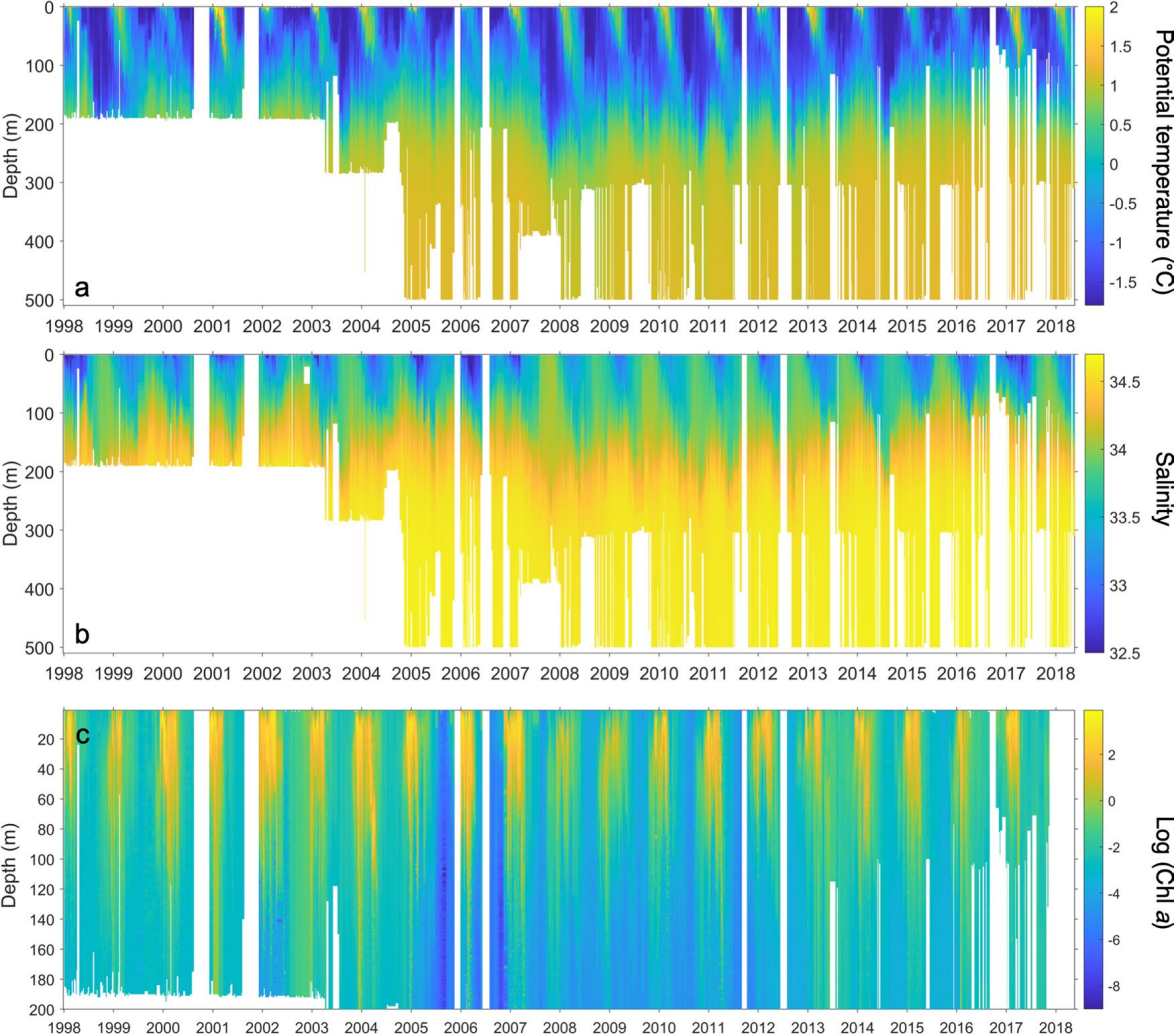
(**a**) Potential temperature, (**b**) Practical salinity and (**c**) Logarithm of total chlorophyll *a* concentration from the RaTS site, measured as part of the RaTS program.

The supply of macronutrients from the mCDW, trace metals from the glacial outflow, shelf sediments and nearshore areas and the strong surface stratification from ice melt (sea ice and glacial ice) mean that conditions are sometimes near optimal for phytoplankton growth^14–17^. Chlorophyll *a* concentrations can exceed 20 mg m^−3^ at the peak of the spring and summer phytoplankton blooms, in contrast to midwinter values well below 0.1 mg m^−3^ (Fig. 5).

The RaTS program has clearly shown the value in sustained observations and sampling in the marginal ice zone on the WAP. The time series has been supported by the US Antarctic Program Palmer Long Term Ecological Research (LTER) study^18,19^, with a near-annual intercomparison station in Marguerite Bay as part of the annual LTER grid along the WAP on the R/V *Laurence M. Gould*, for calibration purposes (see below). The RaTS sampling has captured the variability throughout the annual cycle, made possible due to sampling in the polar winter and spring. In addition, the time series highlights interannual variability, which is influenced strongly by winter sea ice changes and, therefore, the meteorological forcings of that variability^14^. The changes in water mass characteristics from particularly low sea ice years can be seen through the following summer in a range of parameters, and at depth can persist on a decadal timescale when sea ice anomalies are more widespread^14^. The level to which significant decadal variability/trends exist in the data depends on multiple factors, including the nature and forcings of each variable being considered and the level of seasonal and interannual variability of that variable (subject explored in separate publications^14^). The addition of autonomous underwater gliders to the RaTS program has provided an increase in temporal and spatial resolution of physical and bio-optical parameters, and these data are reported separately^11^.

The RaTS program sampling has provided a foundation for other scientific research projects to run alongside the core time-series sampling, for varying periods according to purpose. The additional expertise and equipment that these projects have brought have greatly increased the measurements achieved by the wintering scientists running the time series from Rothera. These projects have been run through BAS core funding, three individual UK Natural Environment Research Council (NERC) research fellowships, Antarctic Funding Initiative, Collaborative Gearing Scheme (CGS) and Collaborative Antarctic Science Scheme (CASS) projects (where BAS hosts visiting UK-based scientists) and a collaboration with the Netherlands Polar Programme. These include (but are not limited to) projects focused on: internal tides and coastal upwelling^20^; inorganic carbon chemistry^21^; macronutrients and trace metals^3,15,16,22,23^; climate active gases^24–26^; sediment trap moorings and export^27–29^; and phytoplankton community dynamics^17,30^.

## Methods

Measurements are led by a wintering scientist (Marine Assistant) based at Rothera Research Station, supported by the boating team and other personnel on station. In the early part of the record, Marine Assistants would typically spend two consecutive winters (spanned by three summers) in Antarctica, though this has since reduced to one winter and two summers. Overlap of personnel in summer allows for training and handover of duties.

**Fig. 6:**
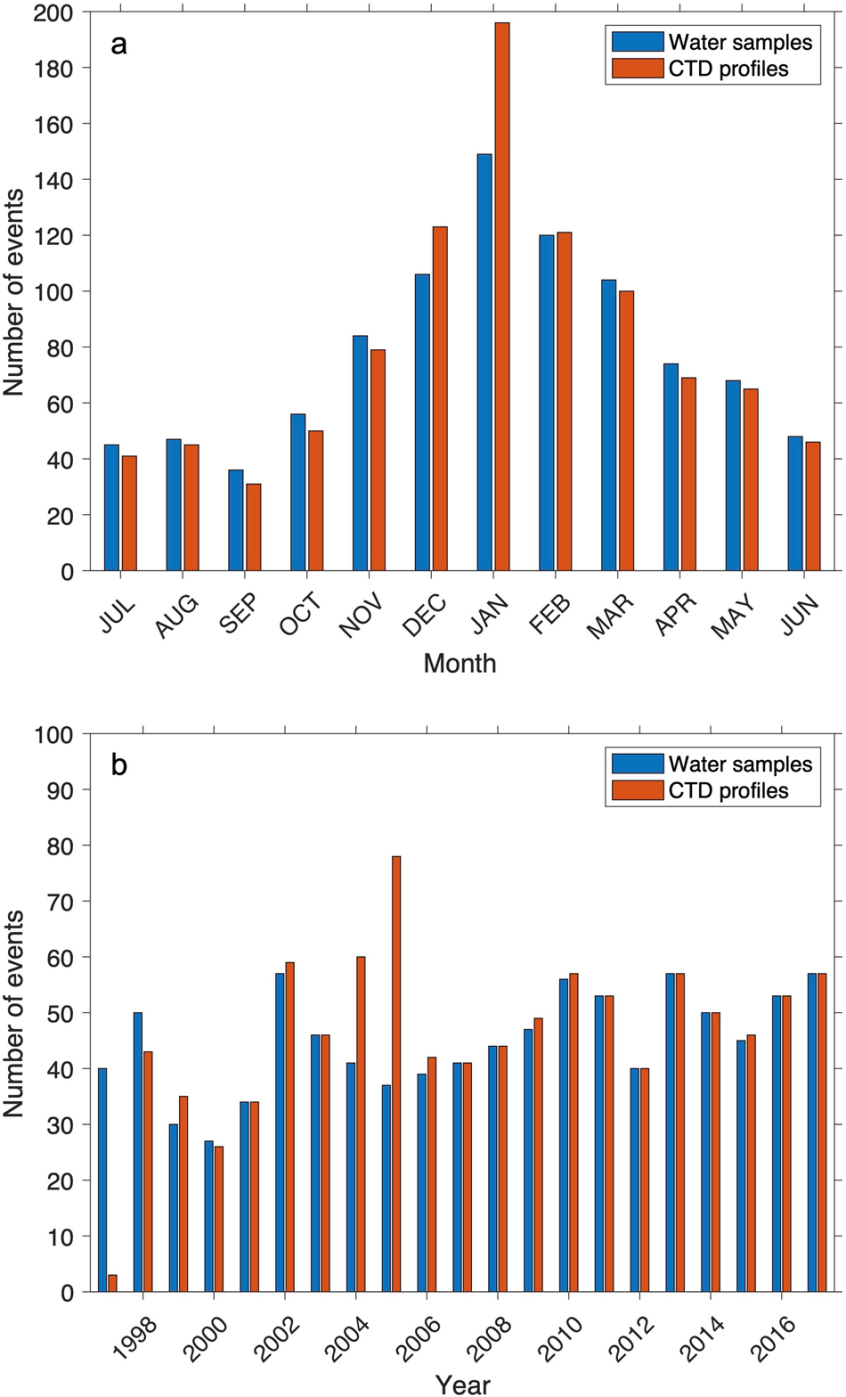
Number of discrete water samples (blue) and CTD profiles (red) carried out as part of the RaTS program plotted against (**a**) month and (**b**) year.

Sampling occurs throughout the year, with typically more events in the austral summer compared with winter due to weather and sea ice conditions (Fig. 6). Most of the sampling is carried out from a small boat with a hand winch. The CTD profiling was carried out to 200 m water depth until 2003, with subsequent profiles carried out for the full depth of the water column, down to 500 m at the primary site (site 1). This change marks the switch from a Chelsea Instruments Aquapak CTD (rated to 200 m) to a pair of SBE19 CTDs rated to 600 m. The CTDs are attached to a Kevlar rope, marked with graded depth markers, and lowered and recovered by a hand winch, with data downloaded in the laboratory at Rothera Research Station after recovery. The CTDs are swapped and calibrated in the UK every other year when possible.

The deepest part of the bay at site 1 is approximately 4 km from Rothera Research Station (Fig. 1), at a water depth of 500 m. Site 2 is 300 m deep and is used when sea ice conditions make site 1 inaccessible, though access through brash ice with a small boat is often achievable, especially with calm conditions and careful boat handling. Some casts are taken close to the wharf to 100 m (site 3) if site 2 is also unreachable. When there is traversable sea ice, the winch is fitted to a sled and towed to one of the sampling points either manually or by skidoo. A hole is then cut in the ice with a chainsaw and the profiling and sample taken as per methods employed from the small boat when fast ice is not present.

The small boat and CTD deployment methodology allows for relatively undisturbed sampling close to the surface. When sampling, the boat is slowed and held at a stop over the chosen site to reduce impact on the water being measured. This approach allows the CTD to measure to within 50 cm of the surface given the configuration of the pumping setup. This is in contrast to stopping a large research vessel and using a larger ship-deployed CTD, where structure in the upper 10 m is often destroyed by mixing. Given the large amounts of meltwater and the importance of surface stratification on setting many of the key processes we seek to address, this is an important distinction. The CTD sensors are generally soaked for three minutes at 15 m water depth, except when profiling through sea ice, or other times when the air temperature is low, when the initial soak is at 40 m to remove ice that might have built up on the frame during transit or during first contact with the seawater.

Water is sampled from 15 m using a Niskin bottle, closed by a messenger weight. Other samples are taken directly from just below the surface from over the side of the boat. Water samples have been taken from other depths using a Niskin bottle by these means, but this requires extra time and effort for winching; accordingly, comprehensive profiles of water sample-derived properties within RaTS are rare. After returning to Rothera (journey time up to 45 minutes depending on site and conditions), samples are transferred to the Bonner Laboratory for analysis, or processing/storage before transferring to the UK for analysis.

### Discrete water samples

#### Chlorophyll a and phaeopigment

**Fig. 7:**
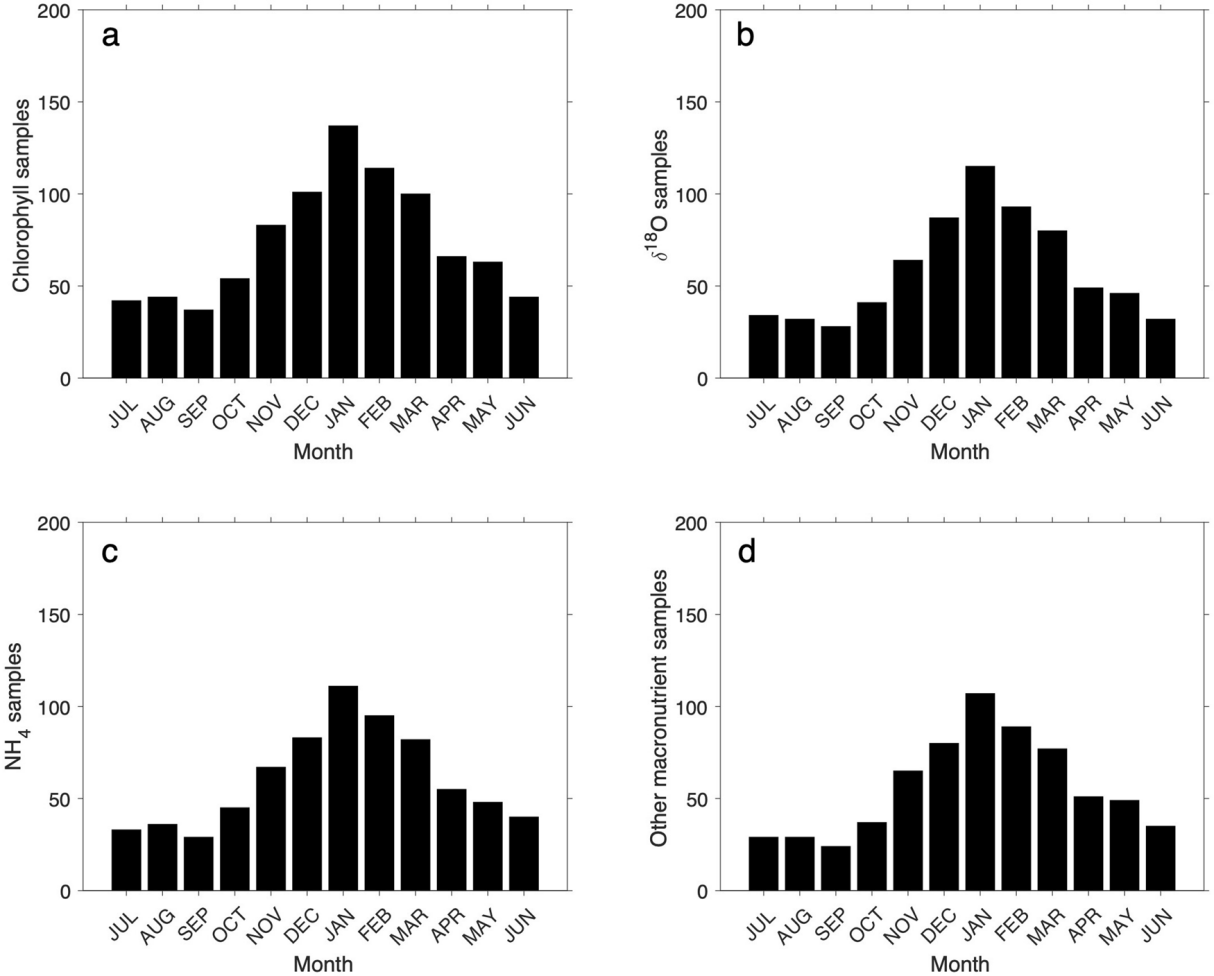
Number of sampling events per month for different parameters at RaTS: (**a**) size-fractionated chlorophyll a; (**b**) *δ*^18^*O* of seawater; (**c**) ammonium; (**d**) other inorganic macronutrients (nitrate, nitrite, silicic acid, phosphate).

**Fig. 8:**
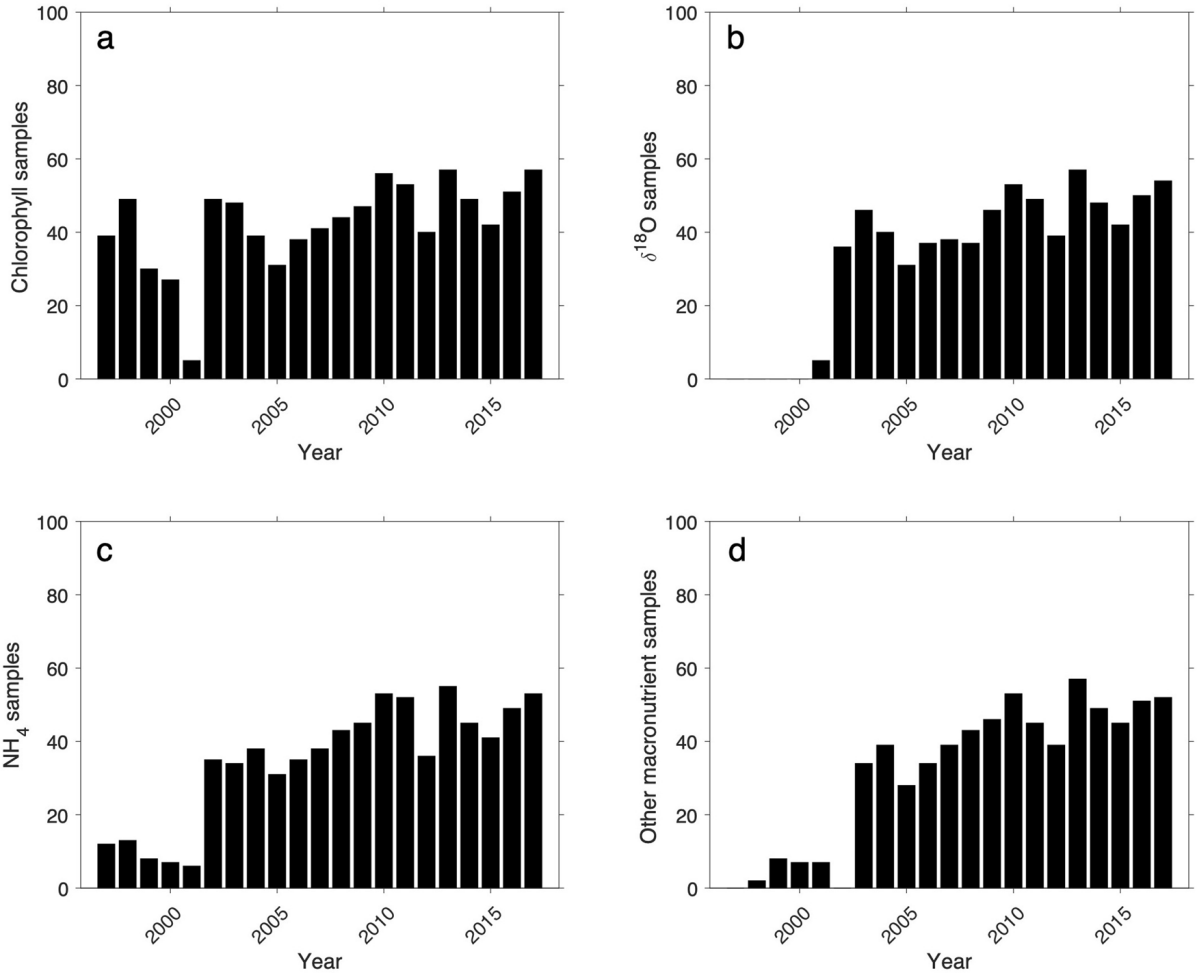
Number of discrete water sample measurements made per year for different parameters at RaTS: (**a**) size-fractionated chlorophyll *a*; (**b**) *δ*^18^*O* of seawater; (**c**) ammonium; (**d**) other inorganic macronutrients (nitrate, nitrite, silicic acid, phosphate).

Collected water samples were mixed gently by inversion, and triplicate samples (100 ml in summer and 500 ml in winter, adjusted for expected chlorophyll *a* concentrations to 250 ml in summer and 2000 ml in winter) were gravity-filtered immediately on return to the research station through sequential 47 mm filters as follows: i) microphytoplankton (>20 *μ*m size fraction via nylon mesh), ii) large nanophytoplankton (5 to 20 *μ*m size fraction via membrane filters), iii) small nanophytoplankton (2 to 5 *μ*m size fraction via membrane filters), iv) picophytoplankton (0.2 to 2 *μ*m size fraction via membrane filters). Pigments were extracted into chloroform/methanol^31^ and measured by fluorometry (Turner AU-10 fluorometer) before and after addition of two drops of 0.1 N HCl under low light levels. Data density of chlorophyll *a* and other discrete water sample measurements are shown in Figs. 7, 8.

#### Inorganic macronutrients

Water samples for the determination of macronutrient concentrations were filtered as soon as possible after collection (GF/C until 2015 and a PALL Acrodisc 32 mm Syringe Filter with 0.8/0.2 *μ*m Supor Membrane and a 60 ml syringe after 2015). Ammonium (measured as ${{\rm{NH}}}_{4}^{+}$) was analysed at Rothera Research Station typically within four hours of collection. From 1997 to 2005 ammonium measurements were carried out using the indophenol technique adapted to utilise dichloroisocyanurate as the chlorine donor and a modified UV incubation^32^. The measurements were calibrated by spiking of triplicate samples^33^. From 2005, ammonium measurements were carried out using ortho-phthaldialdehyde (OPA) and fluorometry^34^. Sample measurements were carried out in triplicate using a Turner TD-700 fluorometer^8^.

Before 2002, other macronutrients (nitrate (${{\rm{NO}}}_{3}^{-}$), nitrite (${{\rm{NO}}}_{2}^{-}$), orthophosphate (${{\rm{PO}}}_{4}^{3-}$) and silicic acid (Si(OH)_4_)) were measured using standard wet chemistry approaches in Rothera Research Station^33^. After 2002, these macronutrient samples were frozen at −20 °C and measured in the UK using a standard nutrient autoanalyser approach^35^. From 2002 to 2017, the samples were measured at the National Oceanography Centre, Southampton (SEAL QuAAtro39 segmented flow auto-analyser); from 2017 onwards, the samples were measured at the Plymouth Marine Laboratory (SEAL analytical AAIII segmented flow colorimetric auto-analyser^36^).

#### Seawater oxygen isotopes

As a tracer measured in tandem with salinity, seawater oxygen isotopes (denoted by *δ*^18^*O*) inform on the relative prevalence of sea ice melt in the water sampled compared with freshwater from meteoric sources (precipitation and glacial melt); it thus has great value in determining the origin of substances delivered to the ocean via the freshwater system^37^. Unfiltered water samples were stored in capped and sealed glass bottles with rubber inserts and minimal head space and kept in the dark at +4 °C during transport to the UK^37^. The samples were measured for *δ*^18^*O* using the CO_2_ equilibration method for oxygen^38^ in triplicate (Natural Environment Research Council Isotope Geosciences Laboratory, Keyworth, UK). Before 2012, the *δ*^18^*O* measurements were made with a SIRA 10 mass spectrometer plus Isoprep18 device. From 2012 to 2017, the *δ*^18^*O* measurements were made with an Isoprime 100 mass spectrometer plus Aquaprep device.

**Table 1:** Structure of Rats_CTD_1998_2017_SL.csv and Rats_CTD_1998_2017_SL.nc files, reporting CTD sensor data.

Variable description	Variable name in the file
**Rats_CTD_1998_2017_SL.csv**	
CTD Site (1,2 or 3)	Ryder_Bay_Sampling_site
Event number	EventIndex
Year	Year
Month	Month
Day of the month	DayOfMonth
Water depth	depth_m
Pressure	sea_water_pressure_due_to_sea_water_dbar
Conductivity	sea_water_electrical_conductivity_S_m-1
*In situ* temperature	sea_water_temperature_C
Practical salinity	sea_water_practical_salinity
PAR	downwelling_photosynthetic_photon_flux_in_sea_water_mol_m-2_s-1
Fluorescence	fluorescence
Chlorophyll a	mass_concentration_of_chlorophyll_in_sea_water_mg_m-3
**Rats_CTD_1998_2017_SL.nc**	
CTD Site (1,2 or 3)	Ryder_Bay_Sampling_site
Event number	EventIndex
Year	Year
Month	Month
Day of the month	DayOfMonth
Water depth	depth
	Units = ‘m’
Pressure	sea_water_pressure_due_to_sea_water
	Units = ‘dbar’
Conductivity	sea_water_electrical_conductivity
	Units = ‘S m-1’
*In situ* temperature	sea_water_temperature
	Units = ‘degree C’
Practical salinity	sea_water_practical_salinity
PAR	downwelling_photosynthetic_photon_flux_in_sea_water
	Units = ‘mol m-2 s-1’
Fluorescence	fluorescence
Chlorophyll a	mass_concentration_of_chlorophyll_in_sea_water
	Units = ‘mg m-3’

### Sea ice observations

Direct (human) observations of ice type and coverage are made on a daily basis by the Marine Assistant, with up to three types of ice observed recorded at any one time. Ice coverage is given in tenths. Ice categories are given in Table 5.

## Data Records

### CTD profiles

The Data Records are provided in comma-separated values (.csv) and NetCDF (.nc) formats and are held by the UK Polar Data Centre^39^. The data coverage of CTD casts is shown in Fig. 6. The sensor data are recorded in Rats_CTD_1998_2017_SL.csv and Rats_CTD_1998_2017_SL.nc (Table 1). The derived variables are recorded in Rats_Strat_1998_2017_SL.csv and Rats_Strat_1998_2017_SL.nc (Table 2).

**Table 2:** Structure of Rats_Strat_1998_2017_SL.csv and Rats_Strat_1998_2017_SL.nc files, reporting CTD sensor data and derived physical oceanographic variables from the RaTS program (1997–2017).

Variable description	Variable name in the file
**Rats_Strat_1998_2017_SL.csv**	
CTD Site (1,2 or 3)	Ryder_Bay_Sampling_site
Event number	EventIndex
Year	Year
Month	Month
Day of the month	DayOfMonth
MLD referenced to 0 m	Mixed_Layer_Depth_deltasigma_0p05_m
MLD referenced to 10 m	Mixed_Layer_Depth_deltasigma_0p05_refdepth_10 m_m
Stratification calculated at 10 m depth intervals	Potential_energy_anomaly_stratification_refdepth_10 m_J_m-2
	Potential_energy_anomaly_stratification_refdepth_20m_J_m-2
	Potential_energy_anomaly_stratification_refdepth_30m_J_m-2
	Potential_energy_anomaly_stratification_refdepth_40m_J_m-2
	Potential_energy_anomaly_stratification_refdepth_50m_J_m-2
	Potential_energy_anomaly_stratification_refdepth_60m_J_m-2
	Potential_energy_anomaly_stratification_refdepth_70m_J_m-2
	Potential_energy_anomaly_stratification_refdepth_80m_J_m-2
	Potential_energy_anomaly_stratification_refdepth_90m_J_m-2
	Potential_energy_anomaly_stratification_refdepth_100m_J_m-2
**Rats_Strat_1998_2017_SL.nc**	
CTD Site (1,2 or 3)	Ryder_Bay_Sampling_site
Event number	EventIndex
Year	Year
Month	Month
Day of the month	DayOfMonth
Stratification calculated at 10 m depth intervals	Potential_energy_anomaly_startification_10m_increments
	Units = ‘J m-2’
MLD referenced to 0m	Mixed_Layer_Depth_deltasigma_0p05
	Units = ‘m’
MLD referenced to 10m	Mixed_Layer_Depth_deltasigma_0p05_refdepth_10m
	Units = ‘m’

### Discrete water samples

The Data Records are provided as comma separated variable (.csv; Table 3) and are held by the UK Polar Data Centre^39^, with each record listing the Event number, Date (YYYY-MM-DD), the parameter (and units), and any additional comments. The data coverage of the discrete water samples, and seawater parameters measured from the samples, is shown in Figs. 7, 8.

**Table 3:** Comma separated variable datasets for discrete water sample parameters made as part of the RaTS program (1997–2017).

Parameter	Filename
Ammonium ($N{H}_{4}^{+}$)	ammonia.csv
Silicic acid (*Si*(*OH*)_4_)	silicate.csv
Phosphate ($P{O}_{4}^{3-}$)	phosphate.csv
Nitrite ($N{O}_{2}^{-}$)	nitrite.csv
Nitrate ($N{O}_{3}^{-}$)	nitrate.csv
Seawater oxygen isotopes (*δ*^18^*O*)	oxygen_isotope.csv
Size-fractionated chlorophyll *a*	chlorophyll.csv
Parameter	Readme file
Size-fractionated chlorophyll *a*	chlorophyll_readme.txt

### Sea ice

The Data Records are provided as comma separated variable (.csv; Table 4) and are held by the UK Polar Data Centre^39^, with each record listing the Event number, Date (YYYY-MM-DD), the site, ice observations, and any additional comments. Abbreviations used in the ice observation dataset are shown in Table 5 and in ice_data_readme.txt.

**Table 4:** Comma separated variable datasets for ice observations made as part of the RaTS program (1997–2017).

Parameter	Filename
Ice coverage in South Cove (south of Rothera runway)	ice_data_south_cove.csv
Ice coverage in Ryder Bay	ice_data_ryder_bay.csv
Ice coverage in Hangar Cove (north of Rothera runway)	ice_data_hangar_cove.csv

**Table 5:** Categories of sea ice observations recorded as part of the RaTS program (1997–2017).

Abbreviations are as follows:
C = Clear
G = Grease ice (unconsolidated ice crystals giving the sea surface a grey appearance)
Pn = Pancake ice (predominantly circular pieces of ice up to 3 m in diameter,
with raised rims where individual pieces have ground together in swell)
B = Brash ice (floating ice fragments up to 2 m across, formed from wreckage of other forms of ice, including meteoric ice)
P = Pack ice (individual floes of ice, typically larger than 2 m)
F = Fast ice (sea ice which remains fast, that is attached to, the coast or islands)
NR = Not recorded
Date source abbreviations:
O = ice observed and recorded
I = inferred, i.e., no observation made but status inferred from observations on other dates

## Technical Validation

**Fig. 9:**
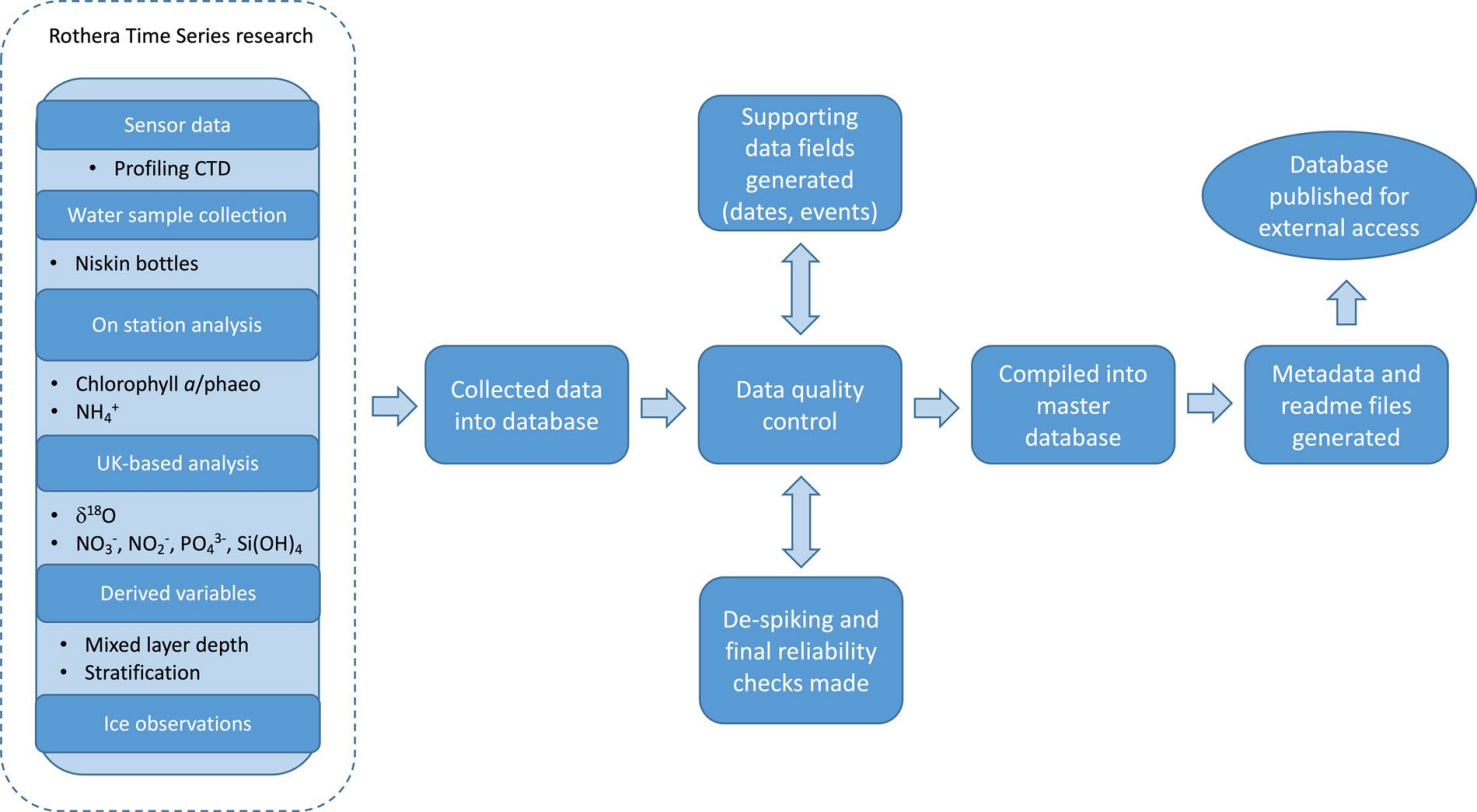
Flowchart of how the RaTS datasets were compiled including collection of sensor data, water sample collection and analyses, data processing and utilisation.

The compilation of the datasets is shown in Fig. 9, with details of the quality control given below.

### CTD profiles

#### Salinity

In polar waters, with temperatures normally below 4 °C, density profiles are largely governed by the shape of the salinity profile. This means that salinity checks can also include density profile checks and the dynamical feasibility of density overturns. There are a limited number of casts with significant density overturns. As these would make the profiles statically unstable (dense water above less dense), in almost all cases they can be ascribed to sensor problems. They can happen throughout a profile but are more common at the surface or bottom of the profile. They were screened by detecting overturns of >0.05 kg m^−3^ and by seeking unusually large deviations between different mixed layer depth calculations (including using the 10 m depth as the reference value). Spikes were then identified and removed manually in salinity in the initial processing. This is usually between 1 and 7 metres of data, though some profiles are completely removed where pump problems make all data invalid. The precision of the salinity data is ensured by discrete samples being collected and by joint casts between the RaTS CTD(s) and that used by the Palmer LTER program, with adjustments applied in initial processing.

#### Temperature

Temperature has little effect on density in the range encountered and is therefore free to vary up and down with depth such that there is no way to ascribe a profile to be physically implausible. The temperature data have been very robust, with no suspicious profiles and very tight matches in all joint casts, it is therefore presented as recorded, except for profiles with pump problems.

#### Photosynthetically active radiation

From 2017 there have been repeating problems with the Photosynthetically Active Radiation (PAR) sensors, despite servicing and replacement. Some values at depth are easily filtered as impossible but other times the values are within bounds but the shape of the profile is implausible. There are standard sampling issues, caused by shading from the boat, ice and varying cloud coverage, such that mean light can increase rapidly with time and/or depth. This makes filtering the problem profiles harder, without removing data where the sensor is working well. Often the shape of the profile is more important than the absolute values, as it is a direct measure of attenuation, so these profiles that increase with depth are of reduced value.

The first filtering is to use a mask created from the first 700 events and remove values <−1. This removes fliers, accounting for the variability driven by weather and attenuation (which can vary considerably with phytoplankton concentration). Away from changing shading/cloud conditions the expectation is for an exponential decline of PAR with depth, and so significant deviations from this can flag up potentially problematic profiles. Estimations of attenuation from PAR profiles are calculated by fitting a regression line to the logarithm of PAR in overlapping 5 m depth intervals down the profile. Checking profiles with negative “attenuation” catches further profiles that are judged to be problematic due to the sensor (rather than natural effects) and these PAR profiles are removed after individual checking. Two profiles in 2015 (Events 1667, 1673) show very unlikely increases at depth. Given the similarities and closeness of the profiles these have also been deemed a sensor problem and blanked out.

#### Mixed layer depth (derived variable)

Quantification of mixed layer depth (MLD) is sensitive to the definition used, which is inevitably a processing choice, with no universally correct answer. Profile-by-profile checking has shown that the use of a 0.05 kg m^−3^ density difference criterion (referenced to either the surface density or density at 10 m) gives a good match to the reduction of chlorophyll with depth, for the period of the year where the mixed layer exceeds the photic zone. This is a good indication of the mixed layer depth that is calculated describing the depth that is, or has very recently been, connected to the surface through vertical mixing.

A time where this has been found to be too tight a criterion is during winter or early spring where spring melt can produce a very shallow layer of fresher, less dense water, above a homogenous zone that has clearly mixed recently. This is exacerbated by our inability to take profiles in the windy conditions that drive the mixing, due to boating safety reasons. To counter this a mixed layer depth relative to 10 m is also calculated, which has use in identifying (for example) deep mixing events that can happen between sampling events.

#### Stratification (derived variable)

There are frequent times through summer where there is strong stratification through the surface depths (to within 5 m, or even 10 cm of the surface) due to the large input of meltwater from sea ice, icebergs and the glaciers. In these circumstances, a mixed layer depth of 1 m or 2 m does little to describe the conditions for mixing of phytoplankton. Due to this, stratification is calculated from the surface to 10 m, 20 m, 30 m through to 100 m in 10 m increments. This is calculated as the additional potential energy that is required to homogenise the depth interval, which gives a physically based metric, with units of J m^−2^ ^40^. This is considered more relevant than a profile of (for example) Brunt Väisälä frequency.

### Discrete water samples

#### Chlorophyll a and phaeopigment

Calibration is carried out twice a year using chlorophyll *a* standards, with samples diluted as required during strong phytoplankton blooms to reduce the range of values measured. The ratio of fluorescence before and after acidification measured each sampling day and is used to assess the reliability of the phaeopigement data. All data are reported as chlorophyll *a* (calculated as total chlorophyll minus phaeopigment^8^). Due to a quality control issue, chlorophyll values after Event 1930 (15 NOV 2017) are currently blanked; it is hoped to incorporate these in a future RaTS data release once resolved.

#### Inorganic macronutrients

Ammonium measurements were carried out in triplicate using a Turner TD-700 fluorometer, and calibrated using standard addition comprising four concentrations, also in triplicate^8^. The detection limit is 0.01 *μ*M. Any negative values were reset at 0.001 *μ*M.

For the other inorganic macronutrients, detection limits are 0.3 *μ*M for nitrate, 0.1 *μ*M for nitrite, 0.2 *μ*M for orthophosphate, and 1.2 *μ*M for silicic acid from 1998 to 2017 (OSIL/NOCS). From 2017 onwards, detection limits for nitrate and orthophosphate were 0.02 *μ*M, and 0.01 *μ*M for nitrite, and 0.02–0.03 *μ*M for silicic acid (PML). The typical uncertainty of the analytical results was between 2 and 3%. Clean sampling and handling techniques were employed during the sampling, processing, defrosting (most critical for the quantitative recovery of silicic acid), and manipulations within the laboratory, and where possible carried out according to the International GO-SHIP nutrient manual recommendations^41^. After 2017, seawater nutrient reference materials (KANSO Ltd. Japan) were analysed to assess analyser performance and for quality control purposes. Spikes were removed from the macronutrient dataset as follows: Nitrite – Events 502 and 504 (APR 2003); Nitrate – Event 328 (15 JUN 2001); Orthophosphate – Event 486 (21 FEB 2003); Silicic acid – Event 314 (18 APR 2001).

#### Seawater oxygen isotopes

Isotope measurements used internal standards calibrated against the international standards VSMOW and VSLAP (before 2012) and VSMOW2 and VSLAP2 (after 2012), with errors typically <0.05‰ for *δ*^18^*O*.

## Data Availability

No custom code was used to generate or process the data described in this manuscript.
